# On the Medicinal Properties of Colchicum

**Published:** 1824-03

**Authors:** 


### Art. III.—*On the Medicinal Properties of Colchicum.* By Dr. Kinglake.

The medicinal properties of colchicum have lately attracted a considerable share of public attention, and the various opinions entertained respecting them are sufficiently interesting to merit particular notice. It is not enough vaguely to affirm that an article of the unquestionable power of colchicum is an efficacious remedy in certain diseases, but it is necessary to ascertain and define the circumstances in which it may be given in the most safe and beneficial manner. The medicinal properties of colchicum reside in an arrangement of its component parts, that produces its narcotic quality. Like digitalis, hyoscyamus, cicuta, belladonna, and other kindred articles, colchicum exerts a strong influence on the sensorial principle of vital power. It affects the action of the nervous system, and, by deranging or diminishing its natural energy, suspends or modifies its susceptibility for being acutely impressed by the usual causes of painful sensation.

It is common to all narcotic agency to impair the natural tone and healthy condition of nervous sensibility. The capacity for being affected when under such influence, is so far reduced as to occasion the sensation of ease where pain had previously existed. It is obvious that this is rather a state of negative sensibility, than a positive removal of morbid pain. It is a consideration of great and anxious moment to determine when, and to what extent, narcotic power should be employed in mitigating the sufferings of disease, which must generally be regarded as an abatement only of morbid violence, and not as a removal of its cause. Painful sensation is commonly rather an effect than the cause of disease, and will therefore not subside until the distempered conditions occasioning the ailment be subdued.

It would at all times be most desirable to afford prompt relief in painful affections, if it were safely and permanently practicable. The narcotic means usually resorted to for this purpose rather induce a temporary unconsciousness of pain, than overcome the disordered action that may have produced it. If the lulling influence of narcotic agency procured a truce with the diseased state, and furnished an opportunity for the resumption of healthy action, it would be a form of remedy that would be most extensively and gratefully applicable. But this is not the fact; it unhappily often permits the morbid state to advance its mischievous process, and covertly to effect an injury that might not have resulted from a more open and direct mode of remedy.

The attempt to cure diseases by narcotics is at best an ambiguous sort of practice, and, if it should not be altogether inert, it is likely to prove deleterious. Its soothing influence is apt to be deceptious, and to be followed by aggravated and more complicated suffering. The objections that are generally applicable to narcotic agency, may be more particularly held against the medicinal properties of colchicum. This medicine has been given of late in various diseases, but the leading indication for its use is to reduce inordinate vascular action, by which the sensorial principle of life is painfully agitated. Its sedative agency has been affirmed to be so far capable of diminishing the contractile power and energy of the heart and arteries, as to incapacitate them for sustaining inflammatory excitement. With this view, it has been administered in various denominations of febrile and inflammatory affection, and in almost every description of painful disease.

Gouty and rheumatic forms of inflammatory excitement have more especially been the objects of its soothing and anti-inflamment powers. Numerous cases have been recorded of the efficacy of colchicum in these affections. Did the narrations that have been given of its remedial powers convey an accurate description of what had actually been effected, the imputation of precipitancy would not be justly incurred in concluding that a specific had at length been discovered for these previously intractable ailments. Deception, however, has here prevailed, and caused the picture of ideal success to be much too highly coloured. An alleviation of general inquietude, and a positive mitigation of local pain, have undeniably resulted from its use, which are often decided advantages; but it may be justly questioned whether its efficacy has reached the morbid cause of the pain,—whether it has corrected the unhealthy deviation that produced the inflammatory state and uneasy feeling, for which it was exhibited.

Independently of vascular depletion by blood-letting, by augmented biliary, pancreatic, urinary, cutaneous, and other secretions, and, above all, by copious discharges from the bowels, it is difficult to conceive what medicinal power can safely reduce vital action, by diminishing the sensorial power and energy of the nervous system. If the object be to cure by debilitating the powers of life, it should be effected by means that may be estimated and measured with the utmost precision, lest a destructive extent of influence be the result. Medicines that impair the strength and efficiency of nervous power are more likely mortally to overwhelm its vital action, than salutarily to repress and regulate its energy. The actions of life and health are happily beyond the reach of being readily injured by substances taken into the stomach. The decomposing and assimilating powers of the alimentary canal supply an effectual barrier against the intrusion into the system of [redacted]-agents; nox but these which possess subtilty of power sufficient to find admission into the frame should be directed thither with the utmost caution, lest the intended herculean remedy should prove a destructive poison. Colchicum is a medicine of decided power, and therefore should be administered under precautions that would be likely at once to insure its beneficial effects, and to obviate all hurtful consequences that may result from it.

Medicines of the narcotic kind operate slowly, and gradually affect the whole system. To overcome the painful and distempered sensibility for which they are usually given, would require that they should be persisted in for a considerable time, during which period effects may be expected to arise that would render a continuance of the medicine unsafe and injurious. The curative object in the use of colchicum, as well as of other narcotics, is to allay morbid pain: the dose that may be necessary to afford this relief may so overwhelm the nervous system, as to induce mental derangement, idiotcy, apoplexy, lethargic torpor, or even paralysis. This would be inflicting the medical evil of rendering the remedy worse than the disease, a mischief never intended, but which unhappily is sometimes incurred by narcotic and other powerful agents, possessing the pervading and disconcerting properties of colchicum.

It is not unusual for narcotics generally, with the exception of opium, after having been largely taken, to concentrate an accumulated or preponderating action on the intestines; in which case, augmented enteric secretions and copious alvine discharges are often the salutary result, in as far as such a determination of narcotic power relieves the brain, and other vital organs, from being hurtfully oppressed or disordered by its influence. The cases in which colchicum has most usefully availed in relieving systematic and local excitement, have been those in which the intestines were cathartically affected by its agency. In other instances, in which less action has been exerted on the intestines, stupor, lassitude, apoplexy, torpor, benumbed sensations, and other symptoms of oppressed and otherwise embarrassed nervous energy, have been the frequent result.

It is difficult to determine the maximum and minimum doses of colchicum. Patients are variously affected by it, according to difference of temperament and habits of vital action. Constitutions that are naturally irritable are better adapted for its salutary agency than those of a more torpid description: in the former, painful sensation may be beneficially relieved; whilst, in the latter, vital action may be lowered to a state of dangerous insensibility.

The range of dose is from five drops to two drachms of either the spirituous or vinous tincture, or of the aqueous infusion, in three table-spoonsful of water, or any other slender vehicle. The medicinal virtues of colchicum are readily yielded to either a spirituous, vinous, or aqueous menstruum. A saturated tincture is obtained by infusing four ounces of the dried root, sliced or bruised, during eight-and-forty hours, in eight ounces of either proof spirit or sherry wine; or an equally efficacious infusion may be procured by digesting, for about twenty-four hours, a similar quantity of the root in the same proportion of boiling water. The root has not been popularly administered in substance, in the form of either powder or pill; and it is probable that the active principle, on which its medicinal efficacy depends, would in that shape be too much encumbered with ligneous matter, to be capable of exerting its power in the direct and specific manner that results from its spirituous and aqueous solutions.

Twenty drops of either the spirituous or vinous tinctures, or of the aqueous infusion, will in general be found to nauseate, and often to move the bowels; though the quantity may be occasionally extended to forty or even sixty drops, without producing any sensible inconvenience. If the purpose for which colchicum is taken be not speedily accomplished,—if the pain that is intended to be subdued by it be not soon overcome, it becomes a subject of considerable importance to determine how long the use of the medicine should be continued. The quantity that may be required to remove the existing pain may occasion either threatening stupor or unappeasable vomiting: when this is the case, either the brain or stomach, and sometimes both, are the situations in which its narcotic agency is

3

more particularly exerted. As in other instances of powerful medicine, such as mercury, digitalis, arsenic, &c. the system becomes sooner or later saturated with colchicum, under its continued use. After this point has been attained, small doses act with much greater power than larger had previously done. This should be an intimation for desisting from its farther use, lest the concentrated violence of its operation should prove seriously injurious. The acme or extent of saturation that should not be exceeded, is shown by small doses inducing nausea, vertigo, loss of appetite, and a sense of great and increasing languor. Under these circumstances, the medicine should be suspended for a week or ten days, to afford an interval for its influence to become gradually dissipated, and finally extinguished, before it would be either prudent or safe to venture on resuming it. If narcotic agency, indicated by diminished or subdued pain, is likely to be lastingly beneficial, this is the time when its salutary effects may be expected to be produced ; and the result will determine the extent of the remedial power that may have been exerted.

The tincture of colchicum, rubbed-in on a joint suffering from rheumatic or any other cause of painful excitement, is said to allay the uneasy sensation, and to affect the general system by absorption. This is possible. A solution of arsenic applied topically, by way of friction, to any part of the cutaneous surface, will be sufficiently absorbed, if persisted in, to induce ptyalism. The skin affords an avenue to the system through the medium of absorption, that may be a commodiously available resource when the stomach, from rejecting irritability or other obstacles, may not admit of powerful remedies being conveyed into the frame through the alimentary canal. Croton oil, tartarised antimony, elaterium, and other medicines, are affirmed to be transmissible by friction, on any part, to the peristaltic power of the intestines, so as to produce a purgative effect, which may, under insuperable impediments in the natural route, be turned to an important account.

Colchicum, like opium and other powerful narcotics, should be sparingly and cautiously directed, and not largely and indefinitely. It should not be forgotten that its operation is rather to induce an inability to feel acutely, than radically to correct the distempered state of sensibility that occasioned the painful affection. Its influence may be regarded as so far torpifying natural susceptibility, as to render it incapable of being painfully impressed, and not as restoring the healthful condition of vital action, in which no unnatural or morbid sensations could exist. Its most beneficial agency seems to consist in furnishing an interval from distempered feeling, during which the inherently restorative powers of life may be enabled to re-instate and adjust the quiescent tone of the natural and undisordered state. If the nervous power from which sensibility naturally emanates, be too much depressed and disturbed, the danger will be, that there will be rather a progressive or dying declension of necessary energy than a salutary repression of its force; in which case, the requisite re-action will not exist, and advancing torpor, decrepitude, and ultimate extinction of life, will be the probable result.

The influence of a medicine capable of such effects should therefore be scrupulously limited and measured, and not inconsiderately persisted in for the purpose of accomplishing a temporary alleviation of pain, at the expence of sinking the powers of life to an irretrievable ebb of depression. It would appear to be more safe and advisable to resort to colchicum, as to the other narcotics, occasionally, to sooth an acuteness of torture, such as sometimes occurs in gouty and rheumatic inflammation, in neuralgia, or tic douloureux, and in every species of painful affection, than to carry it the length of rendering vital power insusceptible of painful impressions.

This highly morbid and fearful state, though of medicinal creation, is full of danger, and may terminate much more disastrously than the disease which it was intended to remove. The point of saturating the system to which some medicines may be safely carried, would be a hazardous and very precarious extent in the use of colchicum. Instances are not wanting of its disabling and paralysing effects; two distressing examples of which have occurred in my own observation. They were both gouty cases, in which a protracted and an unmeasured use of colchicum proved destructive. Stupor of the most unimpressible description arose in the one case; and an incessant vomiting, to the period of dissolution, in the other. It is uncertain what vital organ may be more particularly affected by its agency; whichever may take on a larger portion of its influence than another, to that will be a further determination of its power, when the system is fully saturated with its narcotic effects. With much caution and guarded limitation, it may prove a safe and efficacious auxiliary in allaying painful symptoms of distempered sensibility; but its inconsiderate and undefined administration must be fraught with peril and mischief.

The prevailing practice of indiscriminately resorting to a remedy like that of colchicum, which acts by diminishing or suspending the natural susceptibility for being painfully affected, is too adventurous and doubtful to be warranted on any principle of correct speculation, or of rational safety. The agent that could so far alter the natural conditions of organic feeling as to induce temporary insensibility, may go the unhappy length of annihilating life itself. The transition from the insensible state to that of death is but short, and may be speedily effected by the continued influence of what had occasioned such a morbid reduction of natural feeling.

Gouty and rheumatic excitement, as well as every other description of painful disease, should be brought within safe and tolerably quiet bounds by vascular depletion, diminished temperature, and a lowering and unirritating diet, rather than by an agency such as colchicum affords, that lessens pain by temporarily destroying the power of sensation. The sentient energies of life cannot be immediately depressed without hurtfully enfeebling [illegible] not positively annulling the re-active capability of vital power, on which the reparative processes of health must ultimately depend. Sensation is a most important function in the animal economy, and requires to be duly sustained and regulated, inasmuch as its perversion is disease, its inordinate reduction comparative insensibility, and its irrecoverable depression, or exhaustion, death.

*Taunton; January 10th,* 1824.

