# Errata in Last Number

**Published:** 1824-04

**Authors:** 


ERRATA *in last Number.*

Page 183, line 19, for *nervous* read *noxious.*
— 187, — 11, for *it* read *them.*
— 187, — 11, after the above correction, for *not positively,* read *if not positively.*

